# Significance of re-biopsy for recurrent breast cancer in the immune tumour microenvironment

**DOI:** 10.1038/s41416-018-0197-4

**Published:** 2018-07-23

**Authors:** Koji Takada, Shinichiro Kashiwagi, Wataru Goto, Yuka Asano, Katsuyuki Takahashi, Takaharu Hatano, Tsutomu Takashima, Shuhei Tomita, Hisashi Motomura, Masahiko Ohsawa, Kosei Hirakawa, Masaichi Ohira

**Affiliations:** 1 Department of Surgical Oncology, Osaka City University Graduate School of Medicine, 1-4-3 Asahi-machi, Abeno-ku Osaka, Japan; 2 Department of Pharmacology, Osaka City University Graduate School of Medicine, 1-4-3 Asahi-machi, Abeno-ku Osaka, Japan; 3 Department of Plastic and Reconstructive Surgery, Osaka City University Graduate School of Medicine, 1-4-3 Asahi-machi, Abeno-ku Osaka, Japan; 4 Department of Diagnostic Pathology, Osaka City University Graduate School of Medicine, 1-4-3 Asahi-machi, Abeno-ku Osaka, Japan

**Keywords:** Breast cancer, Cancer microenvironment, Prognostic markers

## Abstract

**Background:**

Immune responses in a tumour microenvironment can be evaluated by analysing tumour-infiltrating lymphocyte (TIL) density; this has been verified in the clinical setting. Although there are many reports on TIL density in primary tumours, little is known about its density in recurrent tumours.

**Methods:**

Of 300 patients treated with neoadjuvant chemotherapy during the study period, 29 were considered for evaluation of TIL density in primary and recurrent tumours. We performed a retrospective analysis of the association between TIL density and prognosis.

**Results:**

TIL density was significantly lower in recurrent tumours than in primary tumours (*P* = 0.007). There was no correlation between post-recurrence survival and TIL density in core-needle biopsy specimens obtained from primary tumours (*P* = 0.837). However, patients with high TIL density in recurrent tumours had significantly better post-recurrence survival than did the corresponding group with low TIL density (*P* = 0.041). Multivariate analysis revealed that high TIL density contributed significantly towards improving post-recurrence survival in all patients (*P* = 0.035; hazard ratio, 0.167).

**Conclusions:**

In recurrent breast cancer, a decrease in TILs density was observed as compared to the primary tumour, and this affects the poor prognosis after relapse.

## INTRODUCTION

An evaluation of the expression of various hormonal receptors is an important component of decision-making for the treatment of breast cancer. However, expression of these receptors may change during treatment and during recurrence.^1–4^ Therefore, when recurrent tumours are diagnosed on histological examination of biopsy specimens, reconfirmation of oestrogen receptor (ER), progesterone receptor (PgR), and human epidermal growth factor receptor-2 (HER2) expression becomes critical.

The immune milieu within the tumour microenvironment (TME) is involved in many anti-tumour treatment effects. For patients with high-risk breast cancers, such as triple-negative breast cancer (TNBC) and HER2-enriched breast cancer (HER2BC), tumour-infiltrating lymphocytes (TILs) are a biomarker for monitoring therapeutic effects and for prognostication. Recently, subset analyses have been initiated to understand the role of TILs. In many studies, TILs are evaluated in pre-treatment specimens, and only clinicopathological features such as pathological complete response (pCR), disease-free survival, and overall survival are considered as end points.^5, 6^ However, little is known about the role of TILs in breast cancer recurrence. Because of discordance in receptor status between primary and recurrent tumours, the TIL profile may vary after treatment; therefore, further evaluation could aid in understanding the role of TILs as a biomarker for treatment effects and prognosis.

We hypothesised that immune responses within the TME may be aggravated at the time of recurrence and may affect treatment of recurrent disease. In this study, we analysed the relationship between changes in TIL density following treatment and post-recurrence survival (PRS) in patients with histologically confirmed recurrence.

## Methods

### Patient characteristics

**Fig. 1:**
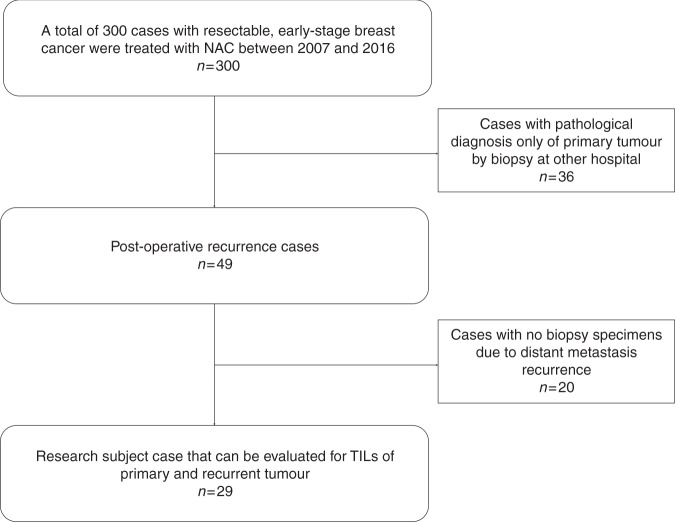
CONSORT diagram. In total, 300 patients with resectable, early-stage breast cancer were treated with neoadjuvant chemotherapy from February 2007 until August 2016 at Osaka City University Hospital; 36 were excluded as the pathological diagnosis of the primary tumour was made at another hospital. Of 264 remaining patients, 49 cases of post-operative recurrence were observed; 20 were excluded due to non-availability of biopsy specimens due to distant metastasis recurrence

A total of 300 patients with resectable, early-stage (stage IIA [T1, N1, M0 or T2, N0, M0], IIB [T2, N1, M0 or T3, N0, M0], or IIIA [T1–2, N2, M0 or T3, N1–2, M0]) breast cancer were treated with neoadjuvant chemotherapy between February 2007 and August 2016 at Osaka City University Hospital.^7^ Thirty-six cases were excluded because the initial pathological diagnosis was made at other hospitals; thus, we were unable to evaluate the pre-treatment TIL status of these patients. Recurrence was observed in 49 of the remaining 264 patients. However, 20 had distant metastases that were not biopsied; hence, 29 cases were examined (Fig. 1).

TNM staging was based on the seventh edition of the American Committee on Cancer Staging Manual.^8^ Breast cancer was confirmed by histological examination of core needle or vacuum-assisted biopsy specimens. Staging was determined using systemic imaging studies, including computed tomography, ultrasonography, and bone scintigraphy. Breast cancer was classified into subtypes according to the immunohistochemical expression of ER, PgR, HER2, and Ki67. Based on their immunohistochemical expression profiles, tumours were categorised into the following immunophenotypes: luminal A (ER+ and/or PgR+, HER2-, Ki67-low); luminal B ([ER+ and/or PgR+, HER2+] or [ER+ and/or PgR+, HER2−, Ki67-high]); HER2BC (ER−, PgR−, HER2+); and TNBC (ER, PgR, and HER2−).

In this study, luminal A and luminal B types were classified as hormone receptor-positive breast cancer (HRBC).^9^ All patients received a standardised protocol of neoadjuvant chemotherapy comprising four courses of FEC100 (500 mg/m^2^ fluorouracil, 100 mg/m^2^ epirubicin, and 500 mg/m^2^ cyclophosphamide) every 3 weeks, followed by 12 courses of 80 mg/m^2^ paclitaxel administered weekly. In addition, patients with HER2BC received trastuzumab weekly (2 mg/kg) or tri-weekly (6 mg/kg) during paclitaxel treatment.^10–12^ All patients received chemotherapy on an outpatient basis. Therapeutic anti-tumour effects were assessed according to the Response Evaluation Criteria in Solid Tumours.^13^

Patients underwent mastectomy or breast-conserving surgery following neoadjuvant chemotherapy.^14^ The pathological effect of chemotherapy was assessed for resected primary tumours after neoadjuvant chemotherapy. The pCR was defined as the complete disappearance of the invasive components of the lesion with or without intraductal components, including that in the lymph nodes, according to the National Surgical Adjuvant Breast and Bowel Project B-18 protocol.^15^ All patients who underwent breast-conserving surgery received post-operative radiotherapy to the remnant breast. Standard post-operative adjuvant therapy was administered based on the subtype. PRS was defined as the time from recurrence to the date of death from any cause. Disease-free survival was defined as the time from surgery to local, locoregional, or distant recurrence, and overall survival was defined as the time from surgery to the date of death resulting from any cause. Progression-free survival was defined as the time from the date of treatment after recurrence to either the date of confirmation of progressive disease or the date of death (whichever came first). The variable *clinical response* was a composite of clinical partial response and clinical complete response, whereas the variable *clinical non-response* was a composite of clinical stable disease and clinical progressive disease; these variables were used to determine the objective response rate.

All patients underwent follow-up physical examination every 3 months, ultrasound every 6 months, and CT and bone scintigraphy annually. The median follow-up interval from the date of surgery was 210 (range, 10–497) weeks for all 264 cases, and was 195 (range, 35–484) weeks for the 29 patients with post-operative recurrence.

### Ethics statement

This study was conducted at the Osaka City University Graduate School of Medicine, Osaka, Japan, according to the REporting recommendations for Tumour MARKer prognostic studies (REMARK) guidelines and a retrospectively written research, pathological evaluation, and statistical analysis plan.^16^ Written informed consent was obtained from all patients. This research adhered to the provisions of the Declaration of Helsinki, 2013. The Ethics Committee of Osaka City University approved the study protocol (Number: 926).

### Histopathological evaluation of TIL density

Core needle or vacuum-assisted biopsy specimens and initial surgical specimens, obtained at the time of breast cancer diagnosis and recurrence, underwent histopathological assessment to determine TIL density; single haematoxylin and eosin-stained tumour sections were examined according to the criteria described by Salgado et al. TILs, defined as lymphocytes infiltrating the tumour stroma, were expressed in proportion to the field investigated; the number of TILs in the stroma surrounding stained cancer cells was quantitatively measured in each field under 400× magnification.^17–19^ Areas of in situ carcinoma and crush artefact were not included. Proportional scores were defined as 3, 2, 1, and 0 if lymphoplasmacytic infiltration of the stroma around the invasive tumour cell nests was >50%, 10–50%, ≤10%, and absent, respectively (Supplemental Fig. 1). The presence of TILs was considered positive when scores were ≥2 and negative when scores were 1 or 0. Two breast pathologists, blinded to clinical information including treatment allocation and outcomes, jointly performed the histopathological evaluation of TIL.

### Statistical analysis

The statistical analyses were conducted using JMP software (SAS, Tokyo, Japan). The relationship between each factor was examined using the *χ* ^2^ test. The Kaplan–Meier method and the log-rank test were used for comparison between PRS and overall survival. The Cox proportional hazards model was used to compute univariate and multivariate hazard ratios (HRs) for the study parameters with 95% confidence intervals (CIs) and was used in a backward stepwise method for variate selection in multivariate analysis. A *P*-value <0.05 was considered significant.

## RESULTS

### Correlation between clinicopathological features and TIL density in primary and recurrent tumours

A total of 300 patients underwent surgery after neoadjuvant chemotherapy; however, in some cases, it was not possible to either evaluate biopsy specimens during diagnosis or perform a biopsy due to distant metastasis. Of the 300 patients, 29 had biopsies performed for recurrent tumours during the post-operative follow-up period (Table 1). All 29 patients were women, with a median age of 53 (30–69) years. Regarding intrinsic subtypes, 11 (37.9%) cases were HRBC, seven (24.2%) were HER2BC, and 11 (37.9%) were TNBC. At initial diagnosis, 15 (51.7%) cases had high TIL density and 14 (48.3%) had low TIL density. Following neoadjuvant chemotherapy, the clinical response and pCR rates were 75.9% and 24.1%, respectively. Regarding adjuvant therapy, 12 (41.4%) patients received hormonal therapy, 14 (48.3%) received radiation therapy, seven (24.1%) received trastuzumab, and five (17.2%) were untreated. The median disease-free survival interval was 65 weeks, and local recurrence was observed in 18 (62.1%) cases; one patient had concurrent bone metastasis.

**Table 1:** Clinicopathological features of 264 patients who were treated with NAC and 29 postoperative-recurrence patients

Parameters	Number of patients who treated with NAC (*n*=264) (%)	Number of postoperative-recurrence patients (*n*=29) (%)
Age (years old)	55 (27–90)	53 (30–69)
Tumour size (cm)	2.9 (1.0–9.8)	2.6 (1.8–8.5)
Lymph node status Negative/Positive	78 (29.5%) / 186 (70.5%)	5 (17.2%) / 24 (82.8%)
Oestrogen receptor Negative/Positive	138 (52.3%) / 126 (47.7%)	19 (65.5%) / 10 (34.5%)
Progesterone receptor Negative/Positive	179 (67.8%) / 85 (32.2%)	20 (69.0%) / 9 (31.0%)
HER2 Negative/Positive	184 (69.7%) / 80 (30.3%)	22 (75.9%) / 7 (24.1%)
Ki67 ≤14% / >14%	87 (33.0%) / 177 (67.0%)	13 (44.8%) / 16 (55.2%)
Intrinsic subtype HRBC/HER2BC/TNBC	129 (48.9%) / 53 (20.1%) / 82 (31.1%)	11 (37.9%) / 7 (24.1%) / 11 (37.9%)
Objective response rate Non-Responders/Responders	26 (9.8%) / 238 (90.2%)	7 (24.1%) / 22 (75.9%)
Pathological complete response Non-pCR/pCR	173 (65.5%) / 91 (34.5%)	22 (75.9%) / 7 (24.1%)
TILs Low/High	140 (53.0%) / 124 (47.0)	14 (48.3%) / 15 (51.7%)

*NAC* neoadjuvant chemotherapy, *HER* human epidermal growth factor receptor, *HRBC* hormone receptor-positive breast cancer (ER+ and/or PgR+), *HER2BC* human epidermal growth factor receptor 2-enriched breast cancer (ER−, PgR−, and HER2+), *TNBC* triple negative breast cancer (ER−, PgR−, and HER2−), *pCR* pathological complete response

Of the 264 patients treated with neoadjuvant chemotherapy, 124 had high TIL density (Supplemental Table 1). Expression of ER (*P* < 0.001) and PgR (*P* < 0.001) was significantly lower in the high-TIL group than in the low-TIL group; HER2 expression was significantly higher (*P* = 0.001). Thus, TIL density was significantly higher in patients with subtypes HER2BC (*P* < 0.001) or TNBC (*P* = 0.011) and was significantly lower in patients with subtype HRBC (*P* < 0.001). The objective response and pCR rates were significantly higher in the high-TIL group than in the low-TIL density group (*P* = 0.031 and *P* < 0.001, respectively).

In the analysis of the 29 patients with post-operative recurrence, those with high, compared with low, TIL density in the primary tumour had a higher objective response rate (*P* = 0.022). However, there was no significant difference in objective response rate based on the density of TIL in recurrent tumours (Table 2). Moreover, no correlation was found between other clinicopathological features and TIL density in either primary or recurrent tumours.

**Table 2:** Correlation between clinicopathological features and TILs in 29 primary and recurrent tumour

	Primary tumour			Recurrent tumour		
Parameters	High TILs (*n*=15)	Low TILs (*n*=14)	*P*-value	High TILs (*n*=6)	Low TILs (*n*=23)	*P*-value
Age at recurrence (years old)						
≤53	7 (46.7%)	8 (57.1%)	0.589	2 (33.3%)	13 (56.5%)	
>53	8 (53.3%)	6 (42.9%)		4 (66.7%)	10 (43.5%)	0.329
Tumour size (cm)						
≤2.6	7 (46.7%)	7 (50.0%)	0.864	2 (33.3%)	12 (52.2%)	
>2.6	8 (53.3%)	7 (50.0%)		4 (66.7%)	11 (47.8%)	0.429
Lymph node status						
Negative	2 (13.3%)	3 (21.4%)	0.5803	1 (16.7%)	4 (17.4%)	
Positive	13 (86.7%)	11 (78.6%)		5 (83.3%)	19 (82.6%)	0.968
Ki67						
≤14%	8 (53.3%)	5 (35.7%)	0.358	1 (16.7%)	12 (52.2%)	
>14%	7 (46.7%)	9 (64.3%)		5 (83.3%)	11 (47.8%)	0.128
Intrinsic subtype HRBC						
Non-HRBC	11 (73.3%)	7 (50.0%)	0.209	2 (33.3%)	16 (69.6%)	
HRBC	4 (26.7%)	7 (50.0%)		4 (66.7%)	7 (30.4%)	0.111
Intrinsic subtype HER2BC						
Non- HER2BC	11 (73.3%)	11 (78.6%)	0.753	6 (100%)	16 (69.6%)	
HER2BC	4 (26.7%)	3 (21.4%)		0 (0%)	7 (30.4%)	0.130
Intrinsic subtype TNBC						
Non-TNBC	8 (53.3%)	10 (71.4%)	0.333	4 (66.7%)	14 (60.9%)	
TNBC	7 (46.7%)	4 (28.6%)		2 (33.3%)	9 (39.1%)	0.803
Objective response rate						
Non-responders	1 (6.7%)	6 (42.9%)	0.022	2 (33.3%)	5 (21.7%)	
Responder	14 (93.3%)	8 (57.1%)		4 (66.7%)	18 (78.3%)	0.571
Pathological complete response						
Non-pCR	10 (66.7%)	12 (85.7%)	0.246	6 (100%)	16 (69.6%)	
pCR	5 (33.3%)	2 (14.3%)		0 (0%)	7 (30.4%)	0.130
Hormone therapy after surgery						
No	8 (53.3%)	9 (64.3%)	0.566	2 (33.3%)	15 (65.2%)	
Yes	7 (46.7%)	5 (35.7%)		4 (66.7%)	8 (34.8%)	0.169
Radiation therapy after surgery						
No	8 (53.3%)	7 (50.0%)	0.864	5 (83.3%)	10 (43.5%)	
Yes	7 (46.7%)	7 (50.0%)		1 (16.7%)	13 (56.5%)	0.087
Trastuzumab after surgery						
No	10 (66.7%)	12 (85.7%)	0.246	6 (100%)	16 (69.6%)	
Yes	5 (33.3%)	2 (14.3%)		0 (0%)	7 (30.4%)	0.130
No-treatment after surgery						
No	13 (86.7%)	11 (78.6%)	0.580	4 (66.7%)	20 (87.0%)	
Yes	2 (13.3%)	3 (21.4%)		2 (33.3%)	3 (13.0%)	0.257
Disease-free survival (weeks)						
≤65	7 (46.7%)	7 (50.0%)	0.864	4 (66.7%)	10 (43.5%)	
>65	8 (53.3%)	7 (50.0%)		2 (33.3%)	13 (56.5%)	0.329
Recurrent tumour site						
Locoregional	8 (53.3%)	10 (71.4%)	0.333	5 (83.3%)	13 (56.5%)	
Other	7 (46.7%)	4 (28.6%)		1 (16.7%)	10 (43.5%)	0.243
TILs of recurrent tumour						
Low	13 (86.7%)	10 (71.4%)	0.329	–	–	
High	2 (13.3%)	4 (28.6%)		–	–	

*TILs* tumour infiltrating lymphocytes, *HRBC* hormone receptor positive breast cancer, *HER2BC* human epidermal growth factor receptor 2-enriched breast cancer, *TNBC* triple negative breast cancer, *ORR* overall response rate, *pCR* pathological complete response

### TIL density in primary vs. recurrent tumours

**Fig. 2:**
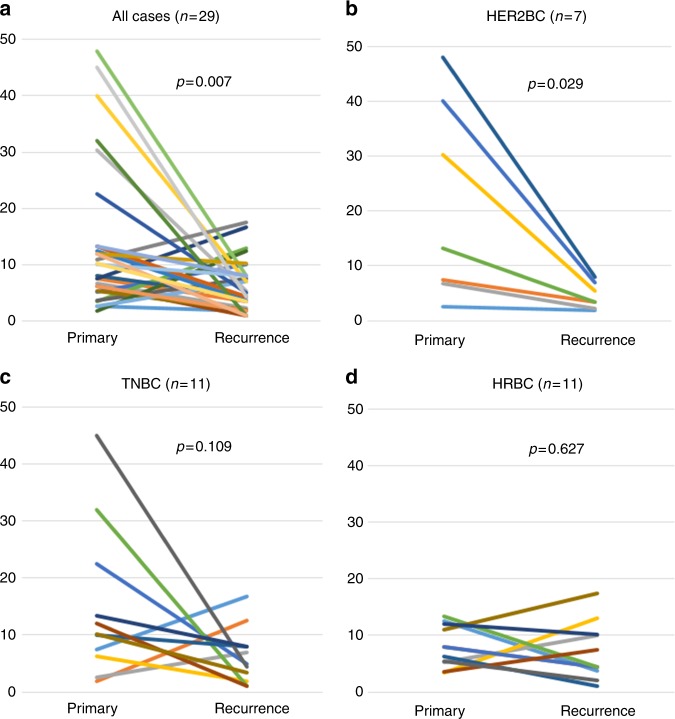
TIL density was significantly lower in recurrent than in primary breast cancers (**a**). There was a significant decrease in TIL density in HER2BC tumours; all recurrent HER2BC tumours demonstrated low TIL density (**b**). No significant change in TIL density was observed in HRBC (**c**) and TNBC tumours (**d**). TIL tumour-infiltrating lymphocyte, HER2BC HER2-enriched breast cancer, HRBC hormone receptor-positive breast cancer, TNBC triple-negative breast cancer

TIL density was significantly lower in recurrent than in primary tumours (*P* = 0.007) (Fig. 2a), specifically in subtype HER2BC tumours (*P* = 0.029); all such tumours had low TIL density (Fig. 2b). Of subtype TNBC tumours, 63.6% (7/11) of primary vs. 18.2% (2/11) of recurrent tumours showed high TIL density (Fig. 2c); no significant difference was observed in recurrent tumours of subtype HRBC (*P* = 0.627) or TNBC (*P* = 0.109) (Fig. 2d).

### Prognostic analysis using TIL density in primary and recurrent tumours

**Fig. 3:**
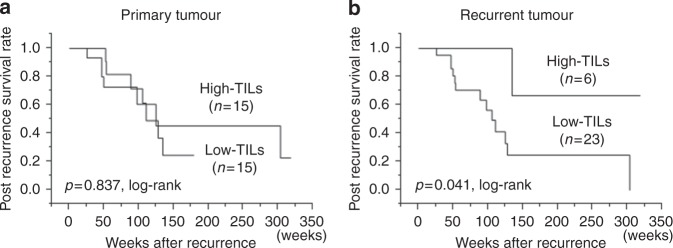
Kaplan–Meier curves stratified by TIL density in primary and recurrent tumours. Post-recurrence survival was similar between patients with high vs. low TIL density in the primary tumour (**a**), but was significantly longer in patients with high (vs. low) TIL density in the recurrent tumour (**b**). TIL tumour-infiltrating lymphocyte

Of the 264 patients whose data were analysed, disease-free survival was significantly longer in the high-TIL group than in the low-TIL density group (*P* = 0.047) (Supplemental Fig. 2a). In particular, a significant difference was observed in patients with subtype HER2BC (*P* = 0.046) and TNBC (*P* = 0.028) tumours (Supplemental Fig. 2b, c); no significant difference was observed for subtype HRBC (*P* = 0.968) (Supplemental Fig. 2d). No significant differences in overall survival were demonstrated between patients with high vs. low TIL density overall or stratified by intrinsic subtype (Supplemental Fig. 3). Although there was no difference in PRS based in the level of TIL density in primary tumours (*P* = 0.83) (Fig. 3a), TIL density in recurrent tumours was significantly associated with PRS. The PRS of patients whose specimens demonstrated high TIL density was significantly better than of those demonstrating low TIL density (*P* = 0.041) (Fig. 3b). In the subtype analysis, the level of TIL density in recurrent tumours was not associated with PRS (HER2BC: unable to calculate, TNBC: *P* = 0.255, HRBC: *P* = 0.063) (Supplemental Figure 4a-c). Progression-free survival was not associated with the level of TIL density overall (*P* = 0.244) or for any of the subtypes (HER2BC: unable to calculate, TNBC: *P* = 0.273, HRBC: *P* = 0.054) (Supplemental Figure 5a-d). On univariate analysis, high TIL density was significantly associated with improved PRS in all patients (*P* = 0.021, HR = 0.154). The multivariable analysis identified high TIL density as an independent favourable prognostic factor (*P* = 0.035, HR = 0.167) (Table 3).

**Table 3:** Univariate and multivariate analysis withpost-recurrence survival

	Univariate analysis			Multivariate analysis		
Parameters	Hazard ratio	95% CI	*P*-value	Hazard ratio	95% CI	*P*-value
Age at recurrence (years old) ≤53 vs. >53	0.874	0.279–2.651	0.811			
Primary tumour size (cm) ≤2.6 vs. >2.6	1.510	0.471–4.632	0.476			
Lymph node status Negative vs. Positive	0.285	0.074–1.370	0.108	0.425	0.110–2.045	0.259
Ki67 ≤14% vs. >14%	0.518	0.158–1.515	0.232			
Intrinsic subtype HRBC No vs. Yes	0.549	0.150–1.649	0.295			
Intrinsic subtype HER2BC No vs. Yes	1.118	0.341–3.265	0.843			
Intrinsic subtype TNBC No vs. Yes	1.736	0.520–5.254	0.350			
Objective response rate Responder vs. Non-responders	0.554	0.179–2.053	0.350			
Pathological complete response pCR vs. Non-pCR	1.589	0.354–5.257	0.504			
Hormone therapy after surgery No vs. Yes	0.599	0.183–1.742	0.351			
Radiation therapy after surgery No vs. Yes	1.066	0.364–3.121	0.906			
Trastuzumab after surgery No vs. Yes	0.908	0.2468–2.743	0.870			
No-treatment after surgery No vs. Yes	2.049	0.456–6.773	0.312			
Disease-free survival (weeks) ≤65 vs. >65	2.714	0.828–12.127	0.103	2.475	0.735–11.227	0.150
Recurrent tumour site Locoregional vs. Other	1.267	0.384–5.684	0.716			
TILs of recurrent tumour Low vs. High	0.154	0.008–0.794	0.021	0.167	0.009-0.900	0.035

*TILs* tumour infiltrating lymphocytes, *HRBC* hormone receptor positive breast cancer, *HER2BC* human epidermal growth factor receptor 2-enriched breast cancer, *TNBC* triple negative breast cancer, *ORR* overall response rate, *pCR* pathological complete response, *CI* confidence intervals

## DISCUSSION

The subtype of recurrent breast cancer may differ from that of the primary cancer. Because this may influence subsequent treatment, re-evaluation at the time of recurrence is necessary. In a study by Dieci et al., subtype changes occurred in 22.7% of cases. PRS was significantly poorer for patients with discordant subtypes than for patients with concordant subtypes.^2^ Furthermore, in one-third of the discordant group, the subtype transformed to TNBC; PRS was poorer in all these cases. In a study by Liedtke et al., the PRS of patients with discordant TNBC was significantly worse than that of patients with concordant TNBC.^3^ In the current study, two cases of HRBC transformed to TNBC: one from HER2BC to HRBC, and one from HRBC to HER2BC. Thus, discordance was observed in 13.8% of cases—half of which transformed to TNBC; there was no difference in prognosis owing to these subtype changes. There was no significant difference between subtypes.^1, 4^ When there were significant differences in PRS among subtypes, more than half of the patients had distant disease recurrence. This could be because TNBC is frequently associated with visceral metastasis, which has a poorer prognosis than local recurrence.^20, 21^ Furthermore, because patients with discordant TNBC had significantly worse PRS than did patients with concordant TNBC, appropriate treatment that considered the change in subtype was probably not implemented. When biopsy was performed earlier for recurrence, as was done in this study, treatment was effective owing to early detection of the subtype change, thereby increasing the probability of choosing the most appropriate treatment modality.

In this study, no difference in PRS was observed, irrespective of biomarker expression in primary or recurrent tumours. However, PRS was reported to be better in ER-positive than in ER-negative breast cancer.^22^ The brain and lungs are also recognised as sites of HER2BC and TNBC recurrence; the fact that recurrent lesions in the brain and lung are only diagnosed on imaging may affect these findings. Moreover, PRS is better for patients with breast cancer, with a longer time to recurrence.^20^ This is probably due to slow proliferation caused by the low malignant potential and proliferative capacity of primary breast cancer cells, with recurrent foci manifesting over a long period. However, no significant differences were observed; further examination of a larger population would be beneficial.

In recent years, the TME has attracted attention as a therapeutic target, and TIL density has been shown to be a useful index for monitoring cancer.^23–25^ TIL density varies depending on cancer subtype. However, many studies examined only pre-treatment specimens, and HER2BC and TNBC are known to express higher levels of TIL than HRBC does.^26, 27^ In the present study, the correlation between clinicopathological features and TIL density in patients who underwent surgery after neoadjuvant chemotherapy was similar; however, in patients with post-operative recurrence, no significant differences based on subtype were observed. This is probably because the frequency of distant metastasis is high in TNBC and HER2BC subtypes, making it impossible to examine tissue specimens of recurrent lesions. Moreover, the post-operative course of some patients with HER2BC and TNBC with high TIL density was good; no disease recurrence was observed. Although this study mostly included cases of local recurrence, a few cases of distant metastasis were also included. In distant recurrence, reduced TIL density has been reported irrespective of the anatomical site, although this was a collective evaluation.^28^

Thus, although no differences in PRS were reported in most subgroup analyses, there was a significant difference in PRS based on TIL density in recurrent tumours. TIL density also influences therapeutic effects after recurrence. Although the number of participants was very small to evaluate PRS for each subtype, a significant decrease in TIL density was observed in recurrent HER2BC; all of these patients demonstrated low TIL density. At the time of recurrence, TIL density was lower not only in subtype HER2BC but also in subtype TNBC.^29, 30^ As described previously, TIL density is often high in subtypes HER2BC and TNBC; these high tumour immune responses contribute to more favourable disease-free and overall survival rates. In the present study, good immune environments in patients who underwent surgery after neoadjuvant chemotherapy led to high objective response and pCR rates. Patients with HER2BC or TNBC with high TIL density had significantly longer disease-free survival durations than did comparative patients with low TIL density. Thus, overall, patients with high TIL density had longer disease-free survival.

The study findings also demonstrate that the immune milieu in the TME of recurrent tumours affects prognosis after relapse. The decrease in TIL density in recurrent tumours indicates immune escape; this contributes to lowering the therapeutic effect. In particular, for patients with HER2BC treated with trastuzumab, immune escape is a factor in recurrence because of the incomplete antibody activity of dependent cellular cytotoxicity. The poor immune milieu in the TME of recurrent tumours could be as a cause of malignancy in HER2BC, indicating that the immune TME is an important contributor to therapeutic effects in recurrence.

There are known differences in the TIL subset between primary breast cancer and distant metastasis or recurrent lesions. Ogiya et al. reported no changes in FOXP3^+^ T-cells, but significantly decreased CD8+ and CD4+ T-cells.^30^ Cimino-Mathews et al. reported that CD8+ and FOXP3+ T-cells significantly decreased.^29^ It is speculated that immune escape occurs with decreases in both the concentrations of CD8+ T-cells (which suppress cancer proliferation) and lymphocytes (which promote cancer proliferation); this may imply an overall decrease in the functioning of the immune system. In these reports, the rates of cancer suppression and promotion were evaluated in lymphocytes. Analysis of TIL subsets and the proportions thereof might be more sensitive as a biomarker. In addition, TILs can be evaluated from any biopsy specimen, irrespective of recurrence, to predict outcomes such as PRS.

In this study, PRS was significantly associated with the level of TIL density in recurrent tumours. Adherence, modification, and proliferation of cancer cells released from cancerous tissues during surgery could trigger recurrence because immune escape is an important factor in tumour progression. Therefore, for patients with recurrent tumours, TIL density could be a biomarker for PRS. A limitation of this study is the small sample size; therefore, it is necessary to conduct further studies to examine subgroup characteristics in a greater number of patients.

In recurrent BC, a decrease in TILs density was observed for recurrent tumours as compared to primary tumours. Furthermore, TILs density in recurrence had an influence on the prognosis after relapse. TILs is also important for post-recurrence therapy, and the possibility that improved TILs density may lead to a better prognosis for relapse patients could also be inferred.

## Electronic supplementary material

COLOUR ARTWORK PRODUCTION FORM

Supplemental table 1

Supplemental Figure 1

Supplemental Figure 2

Supplemental Figure 3

Supplemental Figure 4

Supplemental Figure 5

## Availability of data and material

The data and materials used and analyzed in the current study are available from the corresponding author on request.
