# Supplementary material for: Significance of re-biopsy for recurrent breast cancer in the immune tumour microenvironment
Source: Br J Cancer. 2018 Jul 23;119(5):572–9. doi: 10.1038/s41416-018-0197-4 (PMC6162217; doi:10.1038/s41416-018-0197-4)
Supplement: Supplementary file 1 — COLOUR ARTWORK PRODUCTION FORM [file 41416_2018_197_MOESM1_ESM.docx]

**Supplemental table 1. Correlation between clinicopathological features and TILs in 264 patients who were treated with NAC.**

| Parameters | High TILs (*n* = 124) | Low TILs (*n* = 140) | *p* value |
| --- | --- | --- | --- |
| Age at recurrence (years old)  ≤ 55  > 55 | 65 (52.4 %)  59 (47.6 %) | 68 (48.6 %)  72 (51.4 %) | 0.534 |
| Tumor size (cm)  ≤ 2.9  > 2.9 | 67 (54.0 %)  57 (46.0 %) | 64 (45.7 %)  76 (54.3 %) | 0.179 |
| Lymph node status  Negative  Positive | 38 (30.6 %)  86 (69.4 %) | 40 (28.6 %)  100 (71.4 %) | 0.714 |
| Estrogen receptor  Negative  Positive | 88 (71.0 %)  36 (29.0 %) | 50 (35.7 %)  90 (64.3 %) | <0.001 |
| Progesterone receptor  Negative  Positive | 102 (82.3 %)  22 (17.7 %) | 77 (55.0 %)  63 (45.0 %) | <0.001 |
| HER2  Negative  Positive | 74 (59.7 %)  50 (40.3 %) | 110 (78.6 %)  30 (21.4 %) | 0.001 |
| Ki67  ≤14 %  >14 % | 26 (21.0 %)  98 (79.0 %) | 61 (43.6 %)  79 (56.4 %) | <0.001 |
| Intrinsic subtype HRBC  non-HRBC  HRBC | 87 (70.2 %)  37 (29.8 %) | 48 (34.3 %)  92 (65.7 %) | <0.001 |
| Intrinsic subtype HER2BC  non- HER2BC  HER2BC | 85 (68.5 %)  39 (31.5 %) | 126 (90.0 %)  14 (10.0 %) | <0.001 |
| Intrinsic subtype TNBC  non-TNBC  TNBC | 76 (61.3 %)  48 (38.7 %) | 106 (75.7 %)  34 (24.3 %) | 0.011 |
| Objective response rate  Non-responders  Responder | 7 (5.6 %)  117 (94.4 %) | 19 (13.6 %)  121 (86.4 %) | 0.031 |
| Pathological complete response  non-pCR  pCR | 65 (52.4 %)  59 (47.6 %) | 108 (77.1 %)  32 (22.9 %) | <0.001 |
| Reccurence  Negative  Positive | 107 (86.3 %)  17 (13.7 %) | 108 (77.1 %)  32 (22.9 %) | 0.057 |

TILs, tumor infiltrating lymphocytes. HRBC, hormone receptor positive breast cancer. HER2BC, human epidermal growth factor receptor 2-enriched breast cancer. TNBC, triple negative breast cancer. ORR, overall response rate. pCR, pathological complete response
