# Supplementary figures and images for: Significance of re-biopsy for recurrent breast cancer in the immune tumour microenvironment
Source: Br J Cancer. 2018 Jul 23;119(5):572–9. doi: 10.1038/s41416-018-0197-4 (PMC6162217; doi:10.1038/s41416-018-0197-4)

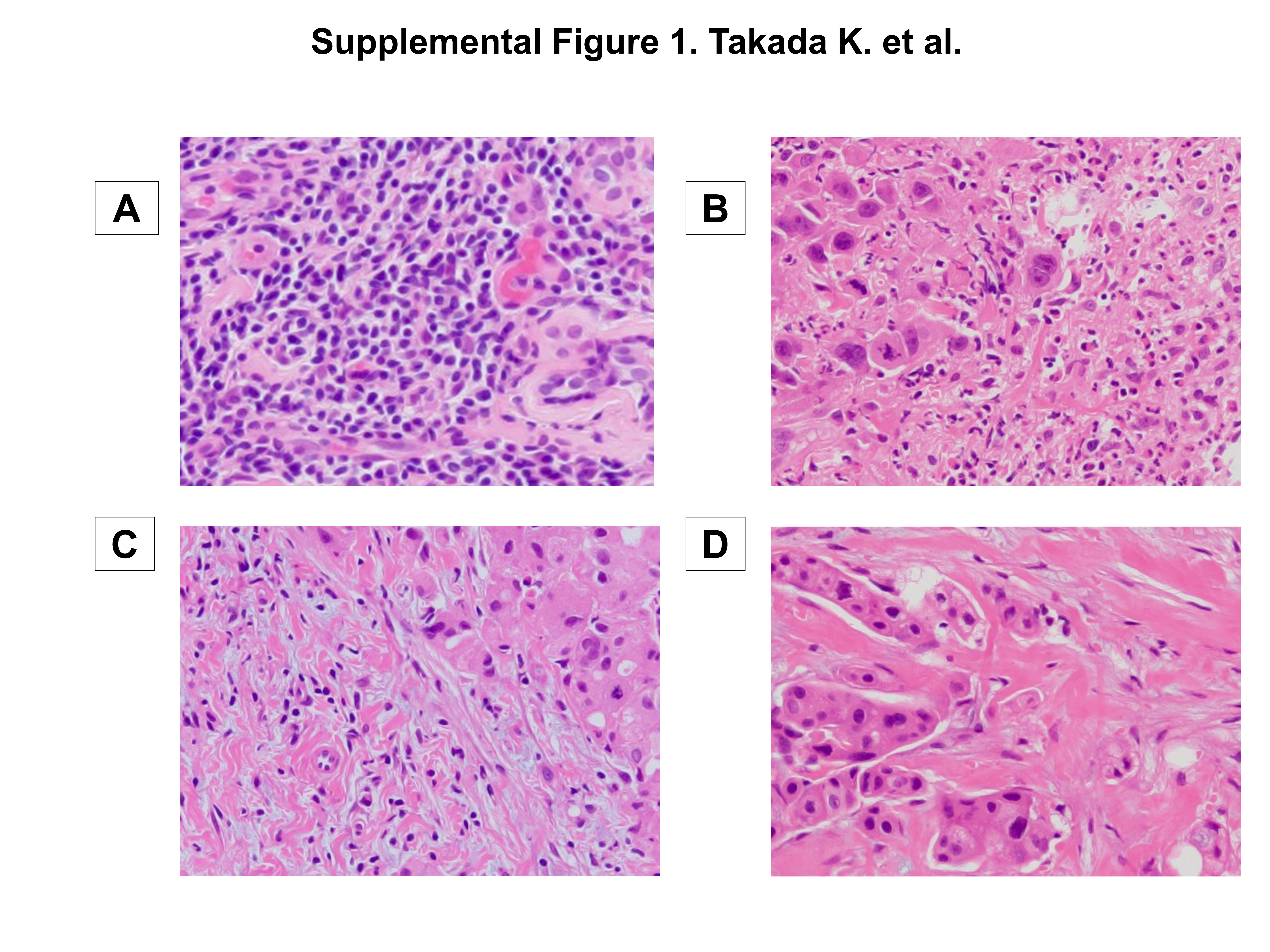

Supplement: Supplementary file 2 — Supplemental table 1 [file 41416_2018_197_MOESM2_ESM.tif]

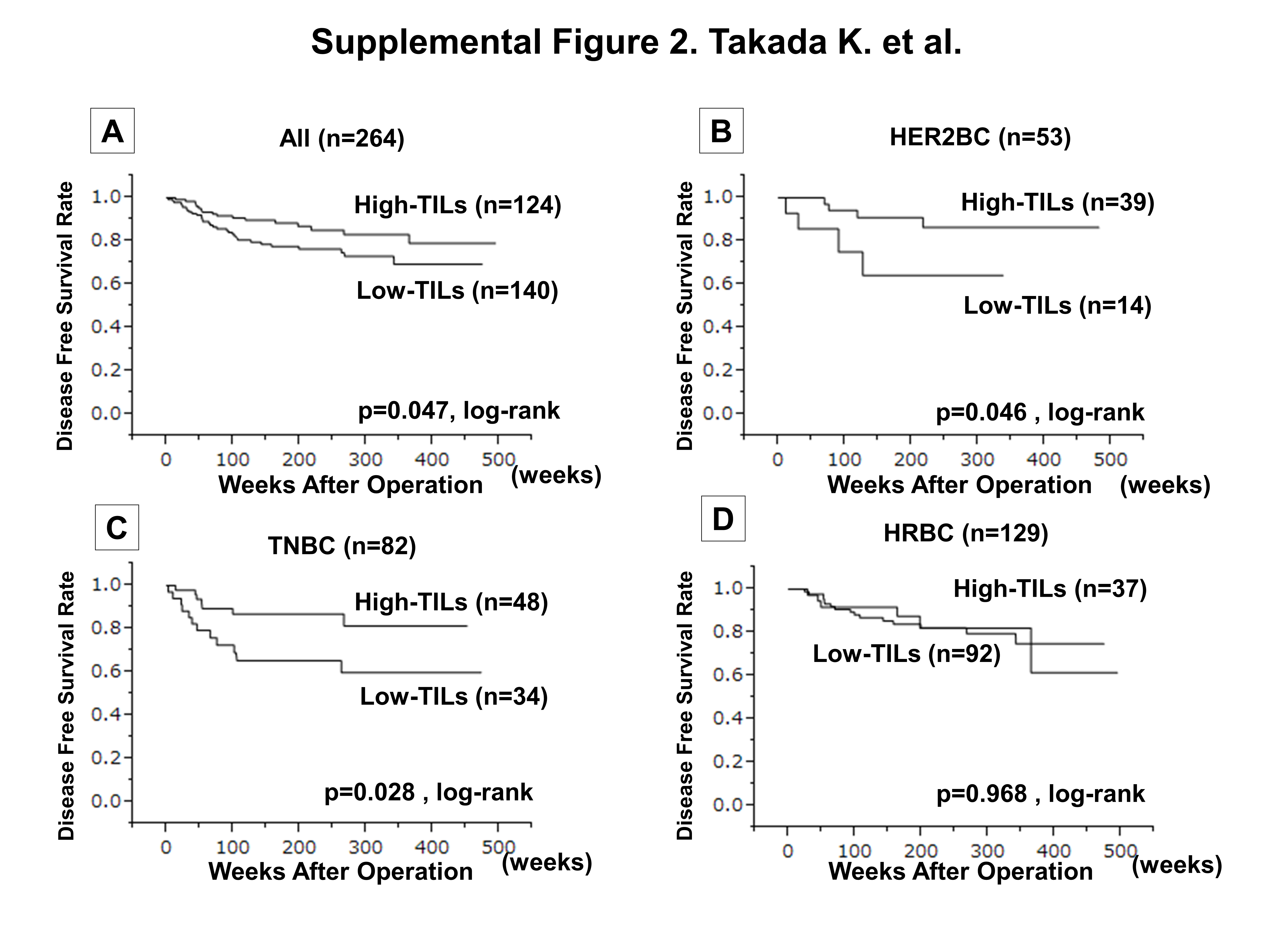

Supplement: Supplementary file 3 — Supplemental Figure 1 [file 41416_2018_197_MOESM3_ESM.tif]

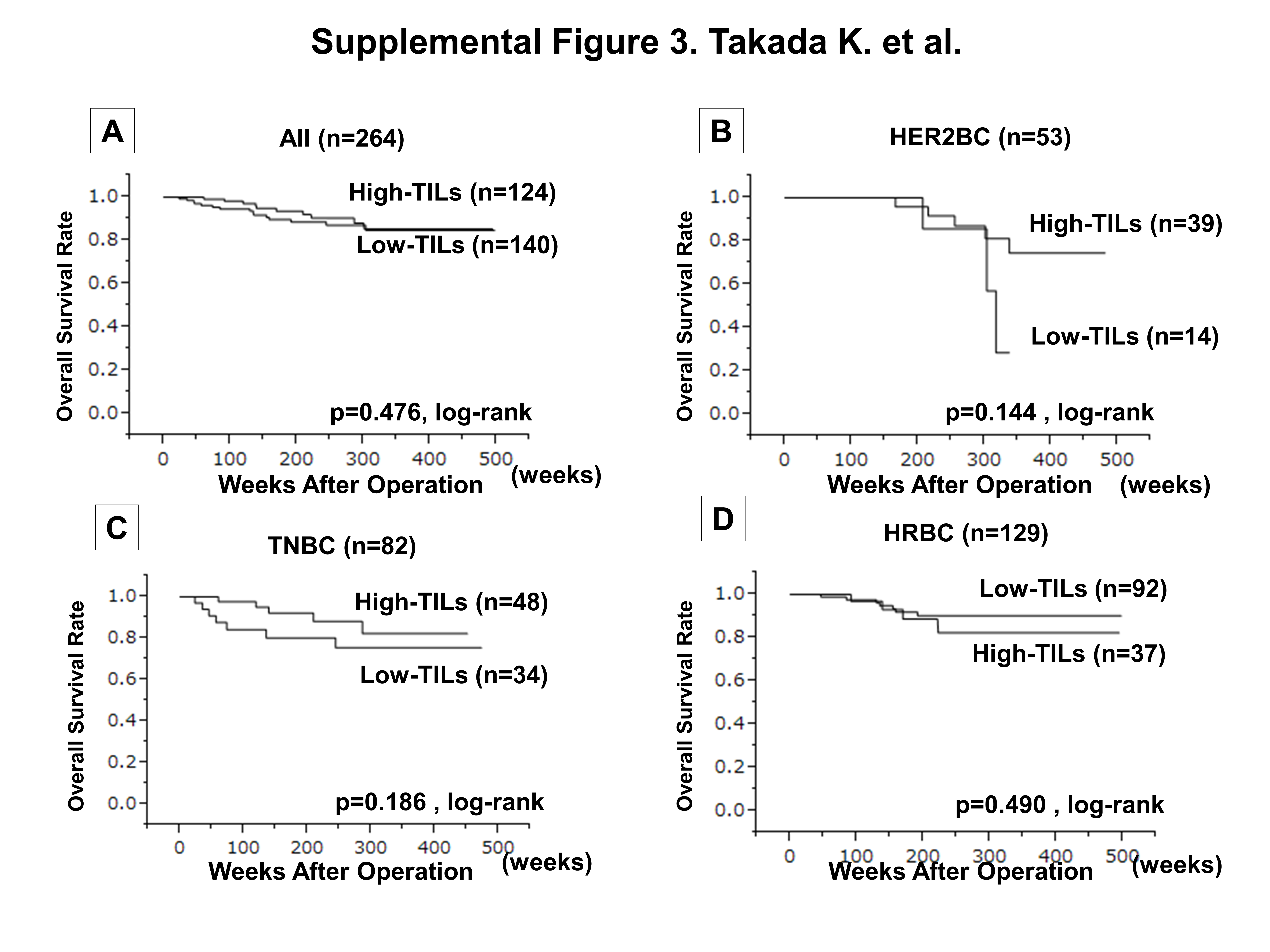

Supplement: Supplementary file 4 — Supplemental Figure 2 [file 41416_2018_197_MOESM4_ESM.tif]

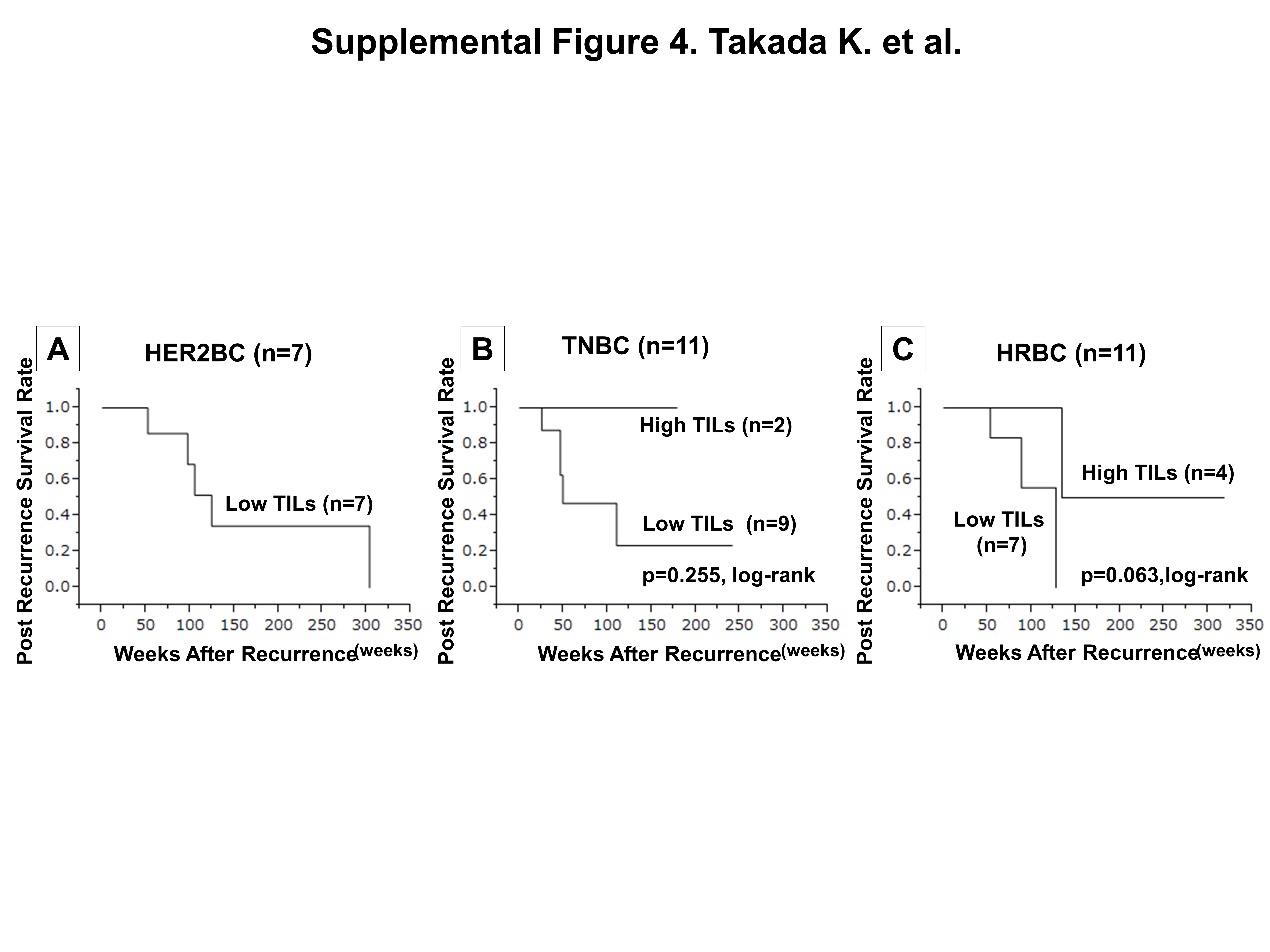

Supplement: Supplementary file 5 — Supplemental Figure 3 [file 41416_2018_197_MOESM5_ESM.tif]

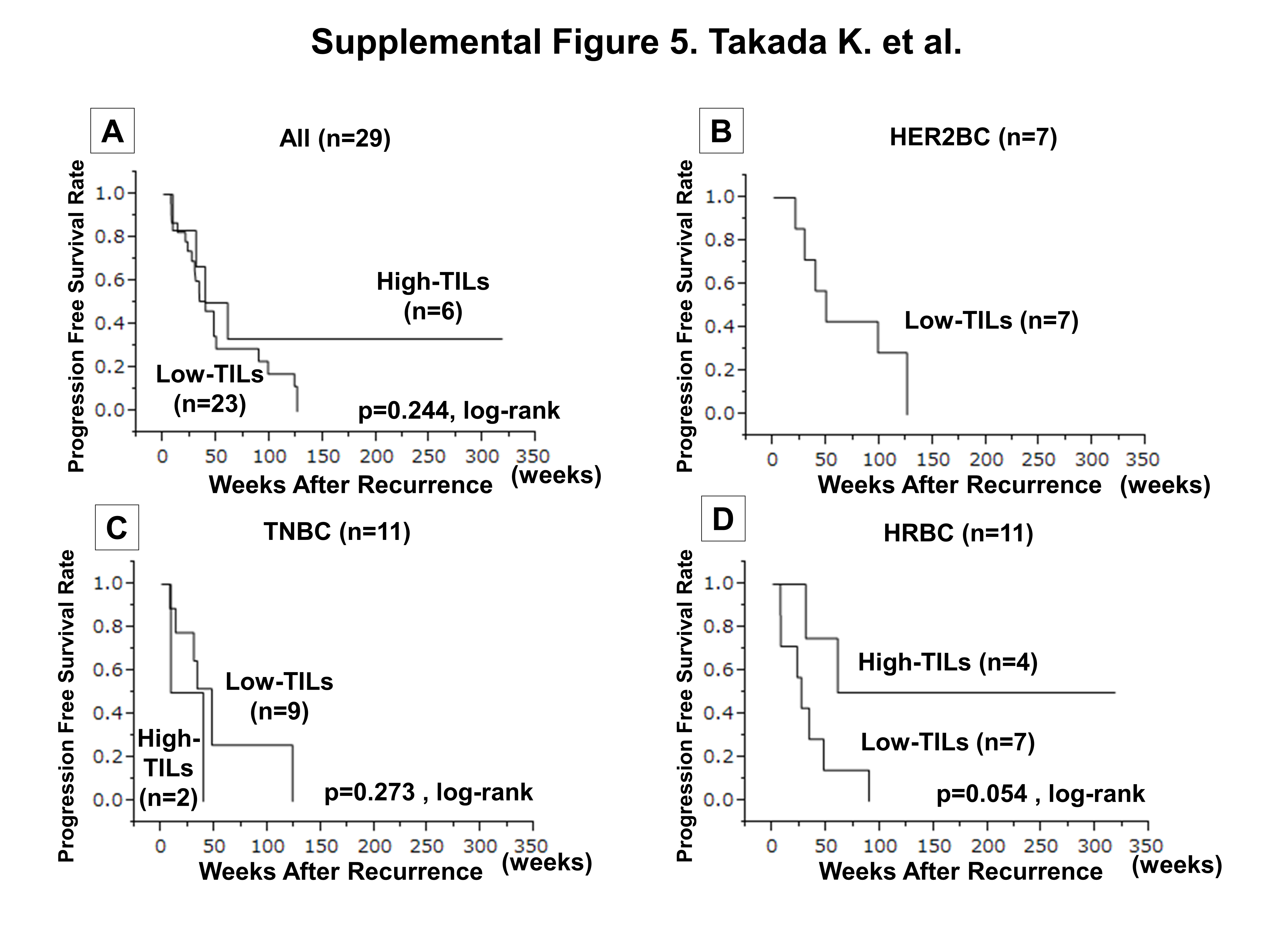

Supplement: Supplementary file 6 — Supplemental Figure 4 [file 41416_2018_197_MOESM6_ESM.tif]
