# Supplementary material for: Significance of re-biopsy for recurrent breast cancer in the immune tumour microenvironment
Source: Br J Cancer. 2018 Jul 23;119(5):572–9. doi: 10.1038/s41416-018-0197-4 (PMC6162217; doi:10.1038/s41416-018-0197-4)
Supplement: Supplementary file 7 — Supplemental Figure 5 [file 41416_2018_197_MOESM7_ESM.docx]

**Supplemental Figure 1.** Histopathological analysis of a single full-face haematoxylin and eosin-stained tumour section showing TIL percentage, defined as the percentage of tumour stroma containing infiltrating lymphocytes. Proportional scores were defined as 3, 2, 1, and 0 if the area of stroma with lymphoplasmacytic infiltration around the invasive tumour cell nests was >50% **(A)**; 10–50% **(B)**; ≤10% **(C)**; and absent **(D)**, respectively.

**Supplemental Figure 2.** Disease-free survival was significantly longer among patients with high, compared with low, TIL density in primary tumours for all breast cancers **(A)** and for subtypes HER2BC **(B)** and TNBC **(C)**. There was no difference in disease-free survival between high and low TIL density in patients with HRBC **(D)**.

HER2BC, HER2-enriched breast cancer; HRBC, hormone receptor-positive breast cancer; TNBC, triple-negative breast cancer.

**Supplemental Figure 3.** Analysis of overall survival, showing no significant difference between patients with high, compared with low, TIL density in primary tumours for all breast cancers **(A)** and for subtypes HER2BC **(B)**, TNBC **(C)**, and HRBC **(D)**.

HER2BC, HER2-enriched breast cancer; HRBC, hormone receptor-positive breast cancer; TNBC, triple-negative breast cancer

**Supplemental Figure 4.** Analysis of post-recurrence survival, showing no significant difference between patients with high, compared with low, TIL density in recurrent tumours for subtypes HER2BC **(A)**, TNBC **(B)**, and HRBC **(C)**.

HER2BC, HER2-enriched breast cancer; HRBC, hormone receptor-positive breast cancer; TNBC, triple-negative breast cancer.

**Supplemental Figure 5.** Analysis of progression-free survival, showing no significant difference between patients with high, compared with low, TIL density in recurrent tumours for all breast cancers **(A)** and for subtypes HER2BC **(B)**, TNBC **(C)**, and HRBC **(D)**.

HER2BC, HER2-enriched breast cancer; HRBC, hormone receptor-positive breast cancer; TNBC, triple-negative breast cancer.
